# Ensemble learning for enhancing critical infrastructure resilience to urban flooding

**DOI:** 10.1038/s41598-025-20970-2

**Published:** 2025-10-22

**Authors:** Yogesh Bhattarai, Vijay Chaudhary, Curtis Walker, Rocky Talchabhadel, Sanjib Sharma

**Affiliations:** 1 Department of Civil and Environmental Engineering, Howard University, Washington, DC 20060 USA; 2 Department of Computer Science and Electrical Engineering, Howard University, Washington, DC 20060 USA; 3 National Center for Atmospheric Research, Boulder, CO 80305 USA; 4 Department of Civil and Environmental Engineering, Jackson State University, Jackson, MS 39217 USA

**Keywords:** Urban flooding, Road networks, Machine learning, Critical infrastructure, Urban resilience, Hydrology, Natural hazards, Civil engineering

## Abstract

Extreme rainfall and flooding severely impact urban systems by disrupting access to critical services, interrupting mobility, and posing challenges for emergency management. Accurate road network flood prediction remains challenging due to complex flow dynamics, coarse-resolution traditional models, and limited data. The main objective of this study is to enhance road-network flood prediction using ensemble machine learning models trained on crowd-sourced flood datasets. Our results for the Washington, D.C. area show that stacked super-ensemble learning improves road flood prediction compared to the voting algorithm and several other base learners, including random forest, support vector machine, bagging, and boosting. Stacking algorithm achieved an accuracy of 0.84, precision of 0.82, and F1-score of 0.82. Shapley additive explanations indicate that elevation strongly influences model prediction accuracy. Stacking ensemble classifies around 5% of road networks as having very high likelihood and 11% as having high likelihood of flooding. We find that over 40% of energy and emergency services are located within high hazard networks. The insights gained from this study can help improve urban flood prediction which is crucial for enhancing community resilience to extreme weather events.

## Introduction

Severe weather events, such as hurricanes, snowstorms, rainfall, and floods, affect billions of people worldwide. The impacts of extreme weather cascade through interdependent and interconnected urban systems, disrupting transportation networks, supply chains, and access to critical services, such as hospitals, schools and power stations, among others^1^. Globally, more than 2 million kms of urban road networks are exposed to 1-in-100-year flood events^2^. In the United States, each year between 2007 to 2016, extreme weather caused over 1.2 million vehicle crashes, 5,000 road deaths, and 400,000 injuries^3^. Road network disruption, even in a specific section, poses a considerable threat to urban resilience, with its impacts rippling across larger spatial domains and persisting for numerous days. Accurate and timely prediction of hazardous road networks can provide decision-makers with richer information to prepare and take prompt action to mitigate potential risks.

Road flooding can stem from poor urban planning. Heavy rain in highly urbanized areas can cause surrounding water bodies to overflow and/or overburden inadequate drainage systems that can lead to localized flooding^4^. In the United States, the Federal Emergency Management Agency (FEMA) produces Flood Insurance Rate Maps that delineate areas considered most vulnerable to riverine and coastal flooding^5^. The most commonly mapped boundaries are the 100-year and 500-year floodplains, representing regions with a 1% and 0.2% annual probability of flooding, respectively. Previous studies have employed multiple approaches for urban flood risk assessment, including FEMA maps to identify high-hazard zones^6^, inundation modeling combined with social vulnerability indices to evaluate flood susceptibility^7^, and the integration of hazard, exposure, and vulnerability to quantify risks to people and property^8,9^. FEMA maps provide valuable information; however they account only for riverine and coastal flooding and overlook pluvial (surface water) flooding^10,11^. Neglecting pluvial flooding can underestimate total flood hazards and risk^12^. Capturing non-linear urban rainfall-runoff processes at the street scale using a physically-based hydrologic-hydrodynamic model is both conceptually and computationally challenging^13,14^. Hence, the accuracy of predictive modeling suffers from a lack of data availability on fine spatial scales, systematic errors, uncertainties, and high computational demand^14–17^. In this study, we tackle these challenges by leveraging ensemble machine learning models trained on crowd-sourced flood datasets.

Flood observation datasets are crucial for improving predictive modeling and designing disaster risk management strategies. Previous studies have shown important applications of high-water marks^18^, satellite observations^6^ and historical flood damage records^7^ to simulate flood risk. Traditional sources, such as high-water marks^19^, are limited to capturing road networks flooding along the river and fail to provide adequate information in areas farther from the river. Existing flood damage records often rely on grid-based damage function that may not accurately represent road-specific impacts^20^. Satellite remote sensing, which, despite its usefulness, often faces challenges in urban areas due to building obstructions and typically provides information after the event rather than in real-time^21^. Crowd-sourced flood datasets provide more detailed and comprehensive insights than traditional sources because they capture localized pluvial flooding at the street scale, offer broader spatial coverage, and reflect real-time ground-level conditions that are often missing from FEMA maps and physically based model outputs^22^. Various crowd-sourced data sets, such as street-level traffic cameras, public webcams, social media posts, news reports, and citizen science initiatives, are readily available and offer more localized, timely insights^23,24^. These diverse data sources have the potential to improve predictive modeling and inform disaster risk management.

Recent advances in computational intelligence and machine learning provide excellent opportunities to enhance data-model fusion, simulate non-linear interactions between key urban processes and predict the spatiotemporal evolution of flood patterns with finer resolution, greater accuracy, and at larger spatial domains, more than ever possible^25^. Emerging techniques, such as using process model outputs to train and optimize machine learning algorithms (emulators or surrogates), embedding machine learning models into physics models (hybrid models), combining individual algorithms to quantify and reduce predictive uncertainty, and using explainable artificial intelligence to better understand model decision-making process offer significant potential to improve predictive accuracy^13,26–28^. Several machine learning algorithms have shown promise to improve road flood prediction, including Random Forest^13,29^, Graph Convolution Networks^30^, and Bayesian networks^31^. However, in complex urban systems where multiple physical processes interact non-linearly, predictions based on a single model are susceptible to errors and bias^32^. In addition, no single algorithm consistently outperforms others in all contexts (e.g. location, training data size, and flood characteristics)^33^.

Ensemble learning can address several limitations of standard machine learning methods, including statistical issues (such as when the search space exceeds the available training data), computational challenges (where the learning algorithm struggles to find the optimal solution), and representation issues (when the algorithms lack suitable fitness functions)^34–36^. Super-ensemble learning is an ensemble technique that trains multiple base learners and provides an optimally weighted average of various base learners^37^. Hence, it can compensate for individual model errors, increase robustness to noise and outliers, and handle intricate complexity of urban features^38–40^. However, the efficacy of these methods has not been systematically evaluated for urban disaster resilience. While previous studies have compared various base learners^41,42^, none have explicitly examined the effectiveness of super ensemble learners in predicting urban road flood risk, particularly using crowd-sourced flood datasets, which have significant potential to improve predictive modeling and inform disaster risk management^38,39,43^.

The key objective of this study is to enhance road flood prediction by using ensemble machine learning models trained on crowd-sourced flood datasets. We evaluate flood hazard to road networks connecting critical urban facilities and employ SHapley Additive exPlanations (SHAP) to interpret the influence of each flood conditioning factor on predictive outcomes. Rather than relying on “black box” models, using models that demonstrate how specific outcomes are achieved can enhance predictive understanding and increase stakeholder confidence in using them for decision-making. We advance existing studies by (i) integrating pluvial flooding into road flood likelihood estimation, (ii) training ensemble machine learning algorithm with crowdsourced flood dataset, (iii) comparing flood likelihood zones with FEMA floodplain maps, (iv) assessing critical infrastructure exposure to flooding, and (iv) leveraging explainable machine learning to identify and understand the most influential factors. The insights gained from this study can provide urban residents and emergency managers with more reliable flood risk information for improved disaster risk management.

## Method and datasets

### Crowdsourced flood database

**Fig. 1:**
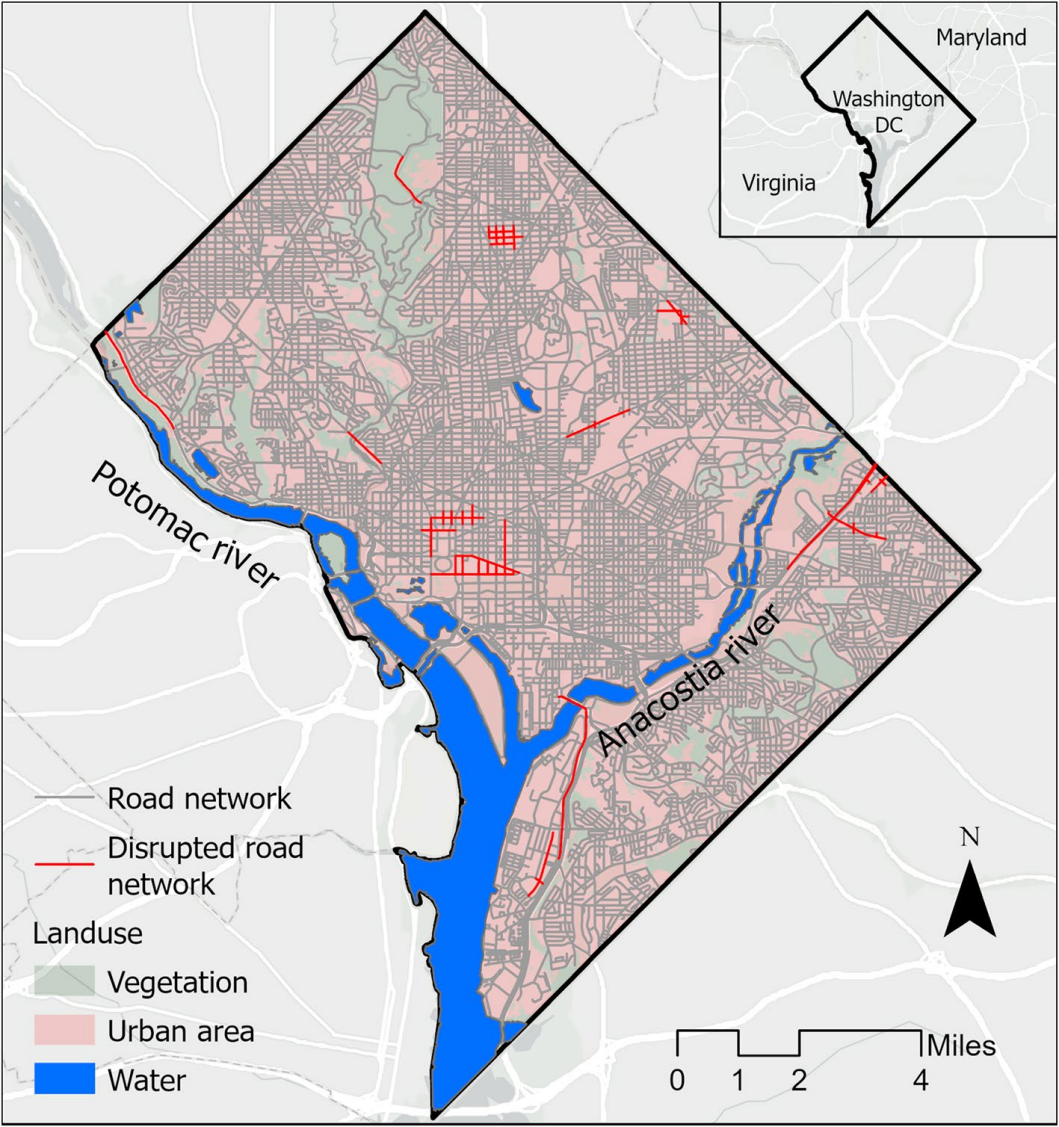
Boundary of Washington District of Columbia (D.C.) with flood-disrupted road networks and different land use. Red networks indicate the disrupted road sections while gray networks indicate non-disrupted sections. Basemap is obtained from ESRI worldmap V2^47^. Map created using ArcGIS Pro^48^.

Washington, D.C., the capital of the United States, is particularly vulnerable to flooding due to the high water levels in the Potomac and Anacostia rivers, as well as its aging and inadequate drainage systems (Fig. 1). The city has experienced several major floods in recent years, including those in 2006 and 2019, which caused significant damage to infrastructure, businesses, and residential areas^44^. The historic rain of 9.41 inches over two days in 2006 caused over $10 million in damage and disrupted several road networks to critical facilities^45,46^. The risk of such destructive events is projected to increase in future with intensifying climate change, expanding urbanization and aging infrastructure^46^.

We compiled information on flooded road networks from local and regional news portals^49^, archived reports^45,50^, and X (formerly Twitter). Using location keywords (’DC’, ’Washington DC’) and flood-related terms (’flood’, ’flooding’, ’road flood’, ’urban flood’, ’flash flood’, ’road closure’, ’rainfall’), we identified flooding dates and affected road locations. We geolocate flooded locations using ArcGIS Pro 3.4.0. We identify 22 locations from 10 rainfall events and found that 150 road networks were impacted by flooding (Fig. 1). Previous studies suggests road networks may be disrupted if it fail to maintain the average traffic speed during extreme events^41,51,52^. We assign ’1’ to the disrupted road networks. To prevent bias toward the majority class, we select 150 non-disrupted road networks. Non-disrupted road networks include roads with no reported flooding in the last five years and maintains normal traffic speed. We utilize the random sample function for selection of balanced dataset of non-disrupted road networks^41^. We assign ’0’ to the non-disrupted road networks.

### Predictors

We employ several flood conditioning factors to account for diverse geospatial characteristics, including hydrologic, infrastructure, topographic, and meteorologic features. The selected input features for machine learning models include elevation, distance to stream, road surface roughness, rainfall, slope, distance to combined sewer outfall, and curve number (Table 1). Elevation represents the difference in height between the grid cells in the digital elevation model^53,54^. The distance to stream is calculated using the Euclidean distance between each cell and the nearest river^41^. Similarly, the distance to combined sewer outfalls is determined using the Euclidean distance between each cell and the nearest outfall. Rainfall estimates are obtained from the National Oceanic and Atmospheric Administration’s Multi-Radar/Multi-Sensor System (MRMS)^55^. MRMS provides near-real-time rainfall estimates by mosaicking data from multiple weather radars, satellites, and observation networks on a grid with a horizontal spacing of 1 km updated every 2 min^56^. Road surface roughness, extracted from the National Land Cover dataset^57^, represents a road’s ability to resist water infiltration and facilitate surface runoff. Slope quantifies the rate of vertical change within each cell of the digital elevation model^58^. The curve number quantifies excess precipitation by analyzing cumulative precipitation depth, soil characteristics, land use, and antecedent soil moisture conditions, for runoff estimation^59^. The curve number is obtained from the national soil layer database^60^. We compute the features of each road network segment based on their average values. We select the road segment as the prediction unit. We select specific road sections to ensure fine spatial granularity while maintaining computational efficiency, which allows the model to capture localized variability within urban road networks^61^.

**Table 1:** Description of conditioning factors used in the study.

Conditioning factors	Description	Resolution	Data source
Elevation	Elevation of road networks	10 m	Open Data DC^62^
Distance to stream	Euclidean distances to the streams	10 m	Open Data DC^62^
Distance from the combined sewer outfall	Euclidean distances to the sewer outfalls	10 m	Open Data DC^62^
Slope	Slope of road networks	10 m	Open Data DC^62^
Curve number	Curve Number of road networks surfaces	30 m	National Soil Layer Database^60^
Road surface roughness	Manning’s roughness coefficient of road surface	30 m	National Land Cover Database^63^
Rainfall	Total rainfall observed over 24 hour period during flooding event	1 km	Multi-Radar/Multi-Sensor System (MRMS)^64^

Feature selection reduces dimensionality by identifying the most relevant features. We selected flood conditioning factors based on previous studies^65^ and the multicollinearity test. High collinearity among features can lead to unstable parameter estimation, unreliable models, and weak predictive performance. The selected features are free from multicollinearity, with a variance inflation factor below 5 and a tolerance greater than 0.1^66^.

### Machine learning models

#### Base learners

Random Forest is a supervised classification method that generates multiple decision trees for the prediction model^67^. The generated decision tree is trained on a randomly selected subset of training data. The final output is determined by aggregating predictions from each decision trees. Random Forest produces robust performance even in the presence of noise, outliers and over-fitting^13,27,68^.

Support Vector Machine is a supervised machine learning technique based on the statistical learning theory and structural risk minimization principle^65^. The algorithm identifies non-linear decision boundaries in a dataset using non-linear kernel functions. Support Vector Machine uses kernel functions (linear kernel, polynomial kernel, radial basis function kernel, and sigmoid kernel) to transform input data into a higher-dimensional space. It finds a separating hyperplane between two classes with a maximum margin between the data points belonging to two different classes. Points above the hyperplane are classified as +1, while points below are classified as -1. The training points lying on the margins on either side of the hyperplane are called support vectors. Once the decision surface is established, it is used to classify new data points into their respective groups.

**Fig. 2:**
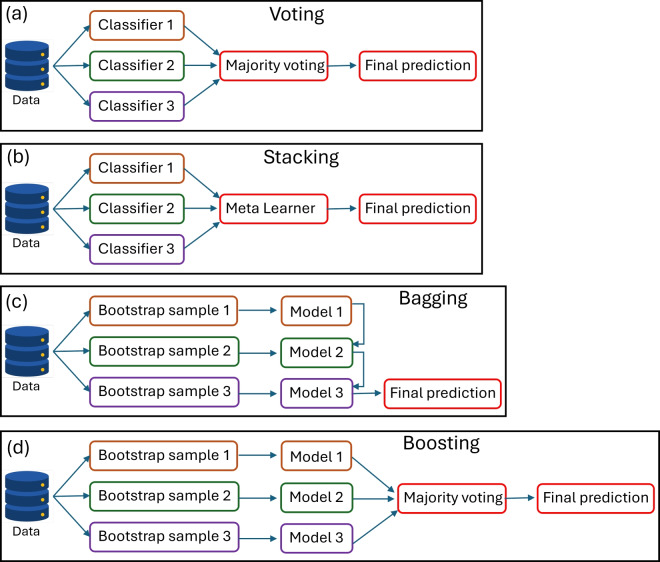
Ensemble learning algorithms: (**a**) Voting, (**b**) Stacking, (**c**) Bagging and (**d**) Boosting.

Bagging trains multiple base models independently and simultaneously on various subsets of training data (Fig. 2c)^67^. Consider a training dataset $S = \{(x_1, y_1), \ldots , (x_n, y_n)\}$, a base machine learning algorithm $L$, and the number of base learners $T$. We follow the ensemble training procedure as: for each $t = 1, \ldots , T$, generate a bootstrap sample $S_t$ from the input dataset $S$ and train a base learner $h_t$ on $S_t$, i.e., $h_t = L(S_t)$. Once all base learners are trained, the outputs are combined to form the ensemble classifier. For a given input $x$, we obtain the ensemble prediction as

1 $$\begin{aligned} H_{\text {Bagging}}(x) = \text {mode}(h_1(x), h_2(x), \ldots , h_T(x)) \end{aligned}$$

where the function $\text {mode}(\cdot )$ selects the most frequent prediction among the base learners. Thus, the final output of the ensemble is the classifier *H*(*x*).

Boosting uses multiple *weak* classifiers to build a *strong* classifier (Fig. 2d)^69^. Let us consider a training dataset $S = \{(x_1, y_1), \ldots , (x_n, y_n)\}$, a base machine learning algorithm $L$, and the number of boosting rounds $T$. We initialize the sample weights $w_i = \frac{1}{n}$ for all $i = 1, \ldots , n$. For each round $t = 1, \ldots , T$, we fit a base learner $h_t$ using the weighted dataset $S$ with weights $w$. We compute the weighted error

2 $$\begin{aligned} \epsilon _t = \frac{\sum _{i=1}^n w_i \cdot {\mathbb {I}}(h_t(x_i) \ne y_i)}{\sum _{i=1}^n w_i}, \end{aligned}$$

where ${\mathbb {I}}(\cdot )$ is the indicator function. We calculate the learner’s weight $\alpha _t = \frac{1}{2} \ln \left( \frac{1 - \epsilon _t}{\epsilon _t}\right)$. We update the sample weights as $w_i \leftarrow w_i \exp (-\alpha _t y_i h_t(x_i))$, and normalize the weights so that $\sum _{i=1}^n w_i = 1$. After $T$ rounds, we compute the final boosted classifier given by

3 $$\begin{aligned} H_{\text {Boosting}}(x) = \text {sign}\left( \sum _{t=1}^T \alpha _t h_t(x)\right) \end{aligned}$$

where $\text {sign}(\cdot )$ returns the predicted class based on the weighted sum of the base learners. Here, we train and test three boosting algorithms: adaptive boosting (AdaBoost), gradient boosting (GBoost) and categorical boosting (CatBoost). AdaBoost focuses on misclassified grids by adjusting their weights. GBoost uses the gradient descent approach for minimizing the overall loss function by fitting new models to the residual of previous models. CatBoost employs gradient boosting to build decision trees sequentially that are efficient in handling categorical features.

We select GridSearchCV from the sklearn library to perform hyperparameter tuning of the base learners^70^. We define a distinct hyperparameter search space for each base learner model. For instance, tuning of Random Forest involves the number of trees (n_estimators) and tuning of Support Vector Machine focuses on the penalty parameter (C) and kernel types. Likewise for the AdaBoost model, we optimize the number of estimators (n_estimators), learning rate, and the depth of the base DecisionTreeClassifier. For Bagging classifier, we tune the number of estimators (n_estimators), maximum samples drawn from the training set (max_samples), and base decision tree depth, with out-of-bag scoring (oob_score=True) retained for internal validation. Similarly, for the Gradient Boosting classifier, we select the number of estimators, learning rate, maximum tree depth, and minimum samples required for splitting internal nodes (min_samples_split) to tune model. We select F1-score as the scoring metrics and perform stratified 10-fold cross-validation to preserve class proportions across folds. For each algorithm, GridSearchCV iteratively trains and evaluates all parameter combinations and selects the configuration with the highest mean F1-score. We use the best parameters for the individual models and development of super ensemble learners.

#### Super ensemble learner

Voting involves the aggregation of predictions from multiple models for predicting final outputs (Fig. 2a)^71^. We select the soft voting technique which involves combining base models by averaging their class probability scores. Let us consider training data $S = {(x_1, y_1), \ldots , (x_m, y_m)}$ and base learning algorithms *T*. First, lets train base learning algorithms: for $t = 1, \ldots , T$: and fit base learner $h_t$ using *S*. Now, for each classifier outputs probability distribution over classes:

4 $$\begin{aligned} h_t(x) = \left[p_t^1(x), p_t^2(x), \ldots , p_t^K(x)\right] \end{aligned}$$

where *K* is number of classes. Then, we obtain soft voting ensemble classifier as :

5 $$\begin{aligned} H_{\text {Voting}}(x) = \arg \max _{k} \sum _{t=1}^T w_t p_t^k(x) \end{aligned}$$

Here, $p_t^k(x)$ is probability of class *k* predicted by classifier $h_t$ $w_t$ are optional weights for each classifier, meanwhile $w_t = \frac{1}{T}$ for uniform weighting by default.

Stacking is an advanced ensemble method that merges diverse machine learning models. Stacking employs a two-layer architecture: base models first generate predictions through cross-validation (Fig. 2b)^72^. These predictions then feed into a meta-model, such as logistic regression^72,73^ . The meta-model learns from the base models’ errors to reduce prediction uncertainty. This two-phase approach minimizes overfitting while maximizing each model’s strengths. Let us consider a training data $S = {(x_1, y_1), \ldots , (x_m, y_m)}$ and base learning algorithms *T*. First, lets train base learning algorithms: for $t = 1, \ldots , T$: and fit base learner $h_t$ using *S*. Now, we can generate a new dataset: for $i = 1, \ldots , m$: create new instance ${({\tilde{x}}_i, {\tilde{y}}_i)}$, $\tilde{x_i} = [ h_1(x_i), h_2(x_i), \ldots , h_T(x_i) ]^{\text {T}}$. Finally, we train meta-learner: ${\hat{h}}$ on new dataset. We obtain the output as ensemble classifier:

6 $$\begin{aligned} H_{\text {Stacking}}(x) = {\hat{h}}(h_1(x), h_2(x), \ldots , h_T(x)). \end{aligned}$$

We develop two-level stacking ensemble classifier for predicting the disrupted road networks. We use StackingClassifier from sklearn library. Combining multiple diverse, competent models through meta-learning produces more robust predictions than single-model tuning approaches^74^. We prepare the stacking model combining Support Vector Machine (margin-based), AdaBoost (shallow trees, bias-reduction), Random Forest (bagged trees, variance-reduction) to capture different patterns. For the meta-learner, we select LogisticRegression, with ridge regularization and the inverse of regularization strength, C = 0.5. Penalized logistic regression reliably select important views, produce sparser models, and maintain competitive classification accuracy, with computational advantages over alternatives^75,76^. We select lower C to prevent overfitting and ensure no single base learner dominates unless it is consistently more predictive. We train base learners directly on the features from the flood dataset, with each model capturing different aspects of the data distribution. We utilize 10-fold cross validation internally to generate out-of-fold predictions for the meta-learner. After that, we train the meta-learner on predictions from base learners. Meta-learner assigns the weight and combines the prediction from base learners to optimize final output.

### Performance assessment

We compile the best parameters selected for the individual base learners to construct the super ensemble learners. We use 70% of our initial datasets for model training and validation and 30% for the model testing. To maintain the high-level randomness for splitting of training and test datasets, we employ the train_test_split function from the sklearn library^70^. We assess the predictive capabilities of base learners and super ensemble learners using several statistical metrics, including accuracy, Kappa score, recall, precision, F1 score, and Receiver Operating Characteristic Area Under the Curve (ROC AUC)^32^:

7 $$\begin{aligned} \text {Accuracy}&= \frac{\text {TP} + \text {TN}}{\text {TP} + \text {TN} + \text {FP} + \text {FN}} \end{aligned}$$

8 $$\begin{aligned} \text {Kappa}&= \frac{P_o - P_e}{1 - P_e} \end{aligned}$$

9 $$\begin{aligned} \text {Recall}&= \frac{\text {TP}}{\text {TP} + \text {FN}} \end{aligned}$$

10 $$\begin{aligned} \text {Precision}&= \frac{\text {TP}}{\text {TP} + \text {FP}} \end{aligned}$$

11 $$\begin{aligned} \text {F1-score}&= 2 \times \frac{\text {Precision} \times \text {Recall}}{\text {Precision} + \text {Recall}} \end{aligned}$$

where True Positive (TP) represents grids correctly identified as flood-prone areas, True Negative (TN) represents grids correctly identified as non-flood areas, False Positive (FP) represents grids incorrectly classified as flood-prone areas, False Negative (FN) represents grids incorrectly classified as non-flood areas, $P_o$ represents the observed agreement, which is the proportion of times the classifier agrees with the actual classification, and $P_e$ represents the expected agreement, which is the proportion of times agreement would be expected to occur by chance. These metrics range from [0,1], where 0 indicates poor predictive performance and 1 indicates perfect predictive skill. We select the best performing model based on the evaluation metrics represented from equations (7–11). We use predict_proba function from sklearn library to determine the probability estimates from selected machine learning model. We classify the obtained probability into five road network disruption hazard levels: very high, high, moderate, low, and safe (Fig. 5). We utilize the natural breaks method available in ArcGIS Pro for performing the classification^77^. Natural breaks can identify the meaningful groupings by minimizing variance within classes and maximizing variance between classes^78^.

## Results

### Predictive performance

**Fig. 3:**
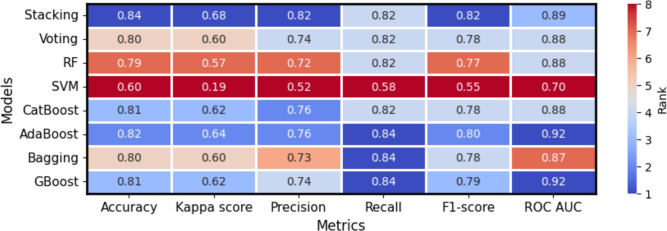
Performance metrics for base learners and super ensemble learning.

Figure 3 depicts the predictive performance of base learners and super ensemble learners. There is no single algorithm that consistently outperforms across all the metrics because each model makes different assumptions, optimizations, and generalizations about the data. Factors such as model complexity, bias-variance tradeoff, data distribution, and feature interactions influence how a model learns and performs. Additionally, certain metrics emphasize specific aspects of performance-such as accuracy, precision, recall, or robustness-leading to variations in evaluation results across models.

We find that stacking ensemble slightly outperforms other models interms of accuracy (0.84), kappa score (0.68), precision (0.82), and F1-score (0.82). AdaBoost, Bagging, and Gradient Boosting demonstrate the highest recall value (0.84). Among boosting algorithms, CatBoost exhibits weaker predictive performance. Furthermore, AdaBoost and Gradient Boosting outperform all other methods in terms of the ROC AUC (0.92). Among the base learners, Support Vector Machine exhibits the lowest predictive performance across all metrics.

**Fig. 4:**
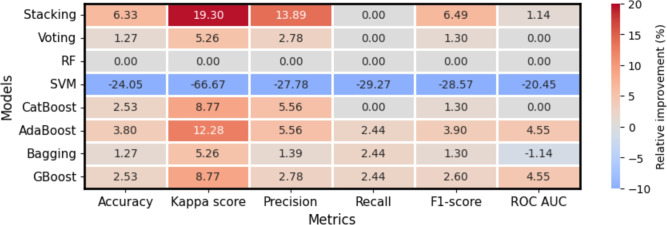
Relative improvement of each predictive algorithm with respect to Random Forest model.

Figure 4 benchmarks the predictive performance of base learners and super ensemble learning with respect to Random Forest. We choose Random Forest as the baseline model for its versatility, robustness, and ability to capture complex relationships while handling outliers and noisy data. Previous studies have also demonstrated the competitive efficiency and accuracy of the Random Forest algorithm in disaster risk prediction compared to other machine learning approaches^13,42,68^. Stacking improves predictive accuracy by 6.33%, Kappa score by 19.30%, Precision by 13.89% and F1-score by 6.49% compared to Random Forest. Similarly, AdaBoost, bagging and gradient boosting method enhance the recall by 2.44%. Adaboost and gradient boosting improves the ROC AUC by 4.55%. However, Support Vector Machine substantially reduces predictive accuracy by 24.05%, Kappa score by 66.67%, Precision by 27.78% and F1-score by 28.57% relative to Random Forest.

### Road flood hazard assessment

**Fig. 5:**
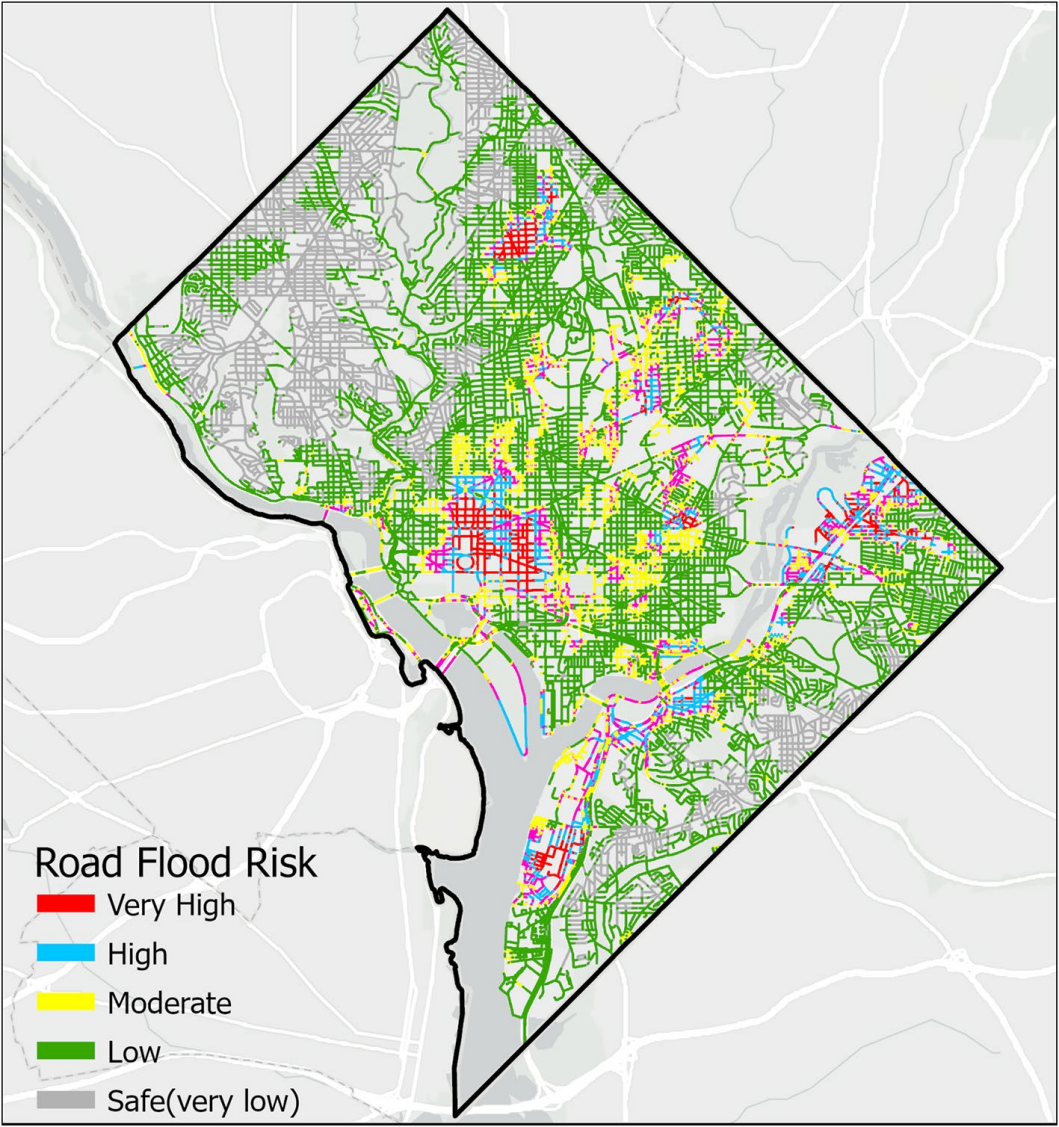
Road network flooding likelihood estimation using stacking model. In the map, gray color indicates safe (i.e., very low), green represents low likelihood, yellow represents moderate likelihood, blue represents high likelihood and red represents very high likelihood road network flooding. Basemap is obtained from ESRI worldmap V2^47^. Map created using ArcGIS Pro^48^.

We employ stacking ensemble learning to predict the probability of road network disruption due to flooding. Overall, 46.72% of the road network falls within the very low-likelihood flooding zone. The remaining network is distributed across the low (22.15%), moderate (14.71%), high (11.21%), and very high-likelihood flooding zones (5.19%). In particular, most of the areas with very high likelihood are concentrated near historical landmarks that have been impacted by recent floodings^49,50^ (Fig. 5).

**Fig. 6:**
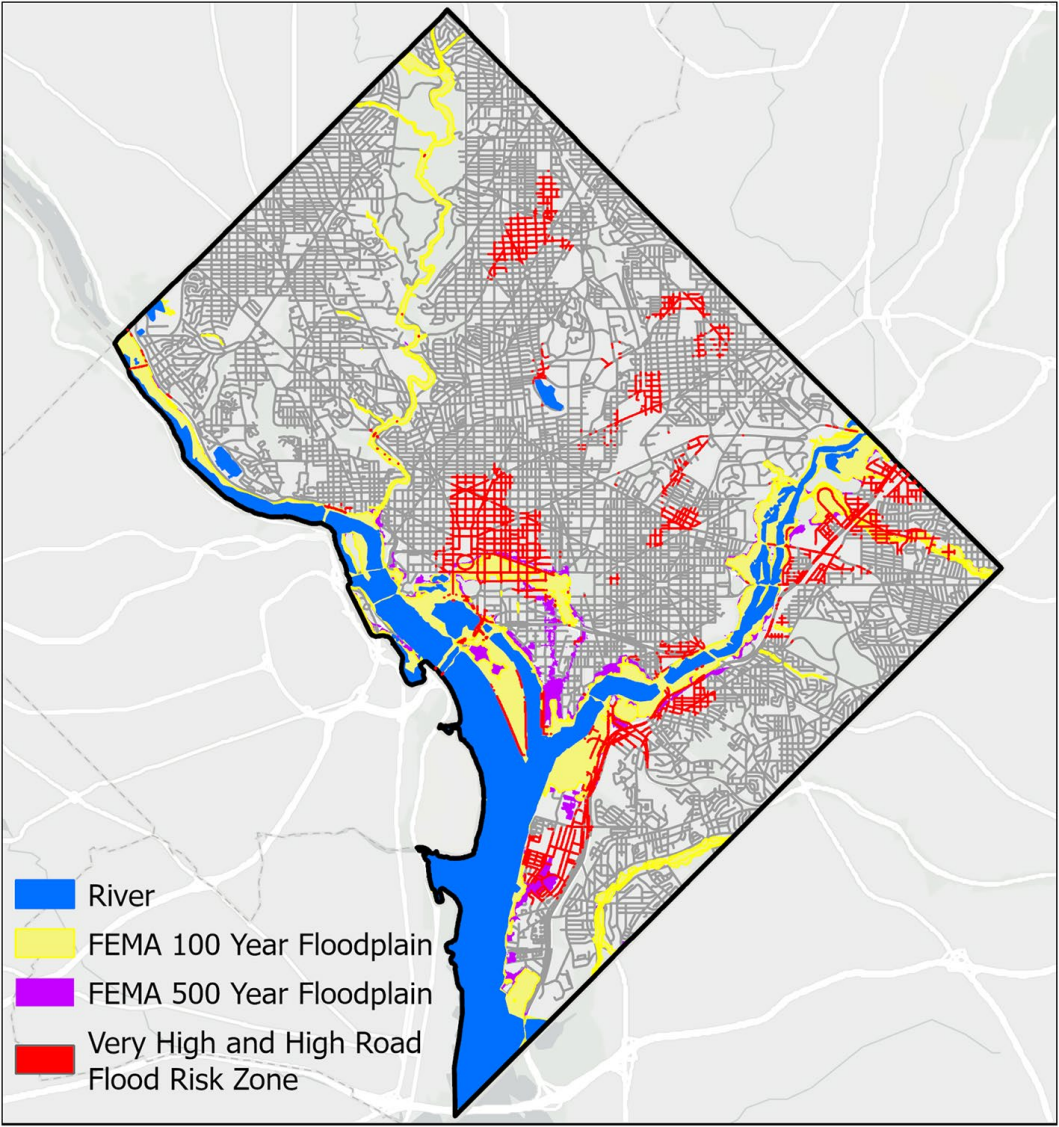
Road flood hazard maps illustrating high and very high flood likelihood zone (in red color) as well as FEMA’s 100-year (in yellow color) and 500-year floodplain (in pink color). Basemap is obtained from ESRI worldmap V2^47^. Map created using ArcGIS Pro^48^.

**Fig. 7:**
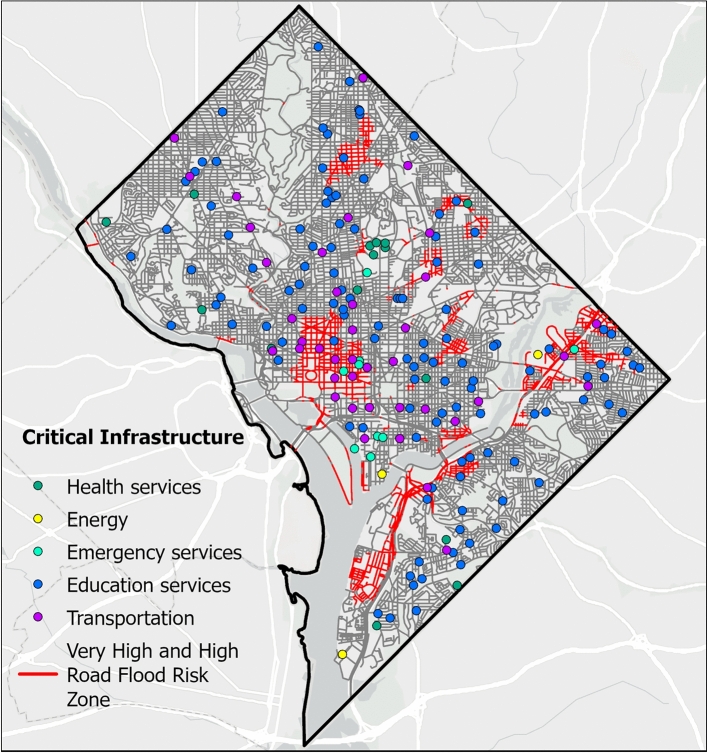
Critical urban infrastructures lying along very high and high road flood likelihood zone. In the figure, health services represent public health institution, energy services represent electrical substations, emergency services represent fire stations, educational services represent public schools and transportation services represent the metro stations. We consider 124 educational services, 9 emergency services, 15 health services, 3 energy services, 40 transportation services, 3 wastewater treatment plants and 573 combined stormwater outlets across the city^79^. Basemap is obtained from ESRI worldmap V2^47^. Map created using ArcGIS Pro^48^.

**Fig. 8:**
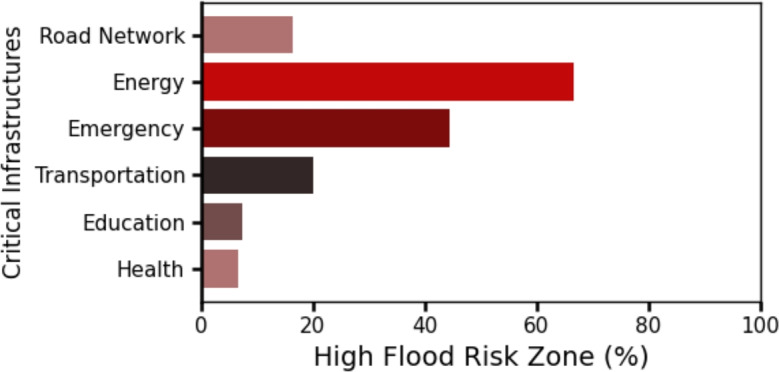
Percentage of critical urban infrastructure within high and very high flood risk zone. In the figure, road network represents all the road segments in Washington D.C. area, health services represents public health institution, energy services represents electrical substations, emergency services represents fire stations, educational services represents public schools and transportation services represents the metro stations.

We compare the high flood likelihood zone with the flood inundation map provided by FEMA (Fig. 6). Our results show that the FEMA flood hazard zone drastically underestimates road flood hazard (Fig. 6). Of the 16.4% of road networks identified within the high flood likelihood zone (Fig. 5), FEMA’s 100-year and 500-year flood zones capture only 12.28% and 19.37%, respectively (Fig. 6). This discrepancy may stem from FEMA’s focus on riverine and coastal flooding and does not account for urban surface flooding occurring away from river channels. We identify critical infrastructures located within high and very high flood likelihood zones (Fig. 7). Overall, 9 out of 124 (7.2%) educational services, 4 out of 9 (44.4%) emergency services, 1 out of 15 (6.7%) health services, 2 out of 3 (66.7%) energy services, and 8 out of 40 (20%) transportation services are exposed to elevated flood hazard (Fig. 8). The concentration of essential services in high flood likelihood areas poses a significant threat to urban mobility safety and can disrupt critical operations during flood event. Educational and emergency services are predominantly located in the city’s center, where government offices and related infrastructure generate high traffic volumes. Flooding along these key routes, as seen during the 2006 and 2019 events^45,49,50^, resulted in widespread disruptions to transportation and essential services. Identifying these critical networks and facilities is crucial for effective planning, resource allocation, and timely interventions in high-risk areas.

### Feature importance

**Fig. 9:**
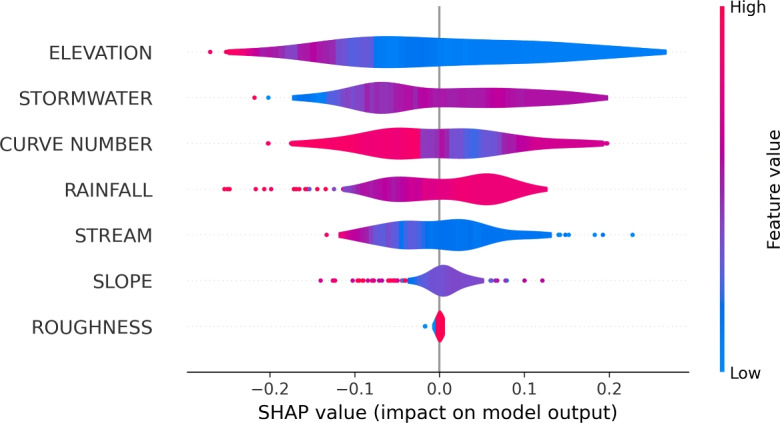
Feature importance to conditioning factors. The larger SHAP value represents feature corresponding to the higher prediction accuracy. In the figure, STORMWATER represents Euclidean distance from the combined stormwater outfall and STREAM represents distance from nearest stream.

Using SHapley Additive exPlanations (SHAP) technique, we quantify each feature’s contribution to the model’s predictive performance by assessing their impact on mean performance metrics^80^ (Fig. 9). SHAP values use cooperative game theory principles to equitably allocate the impact of each feature on model predictions, offering a more mathematically rigorous alternative to conventional feature importance techniques^81^. We select AdaBoost model in SHAP to interpret the contribution of each factor to the model. AdaBoost can reduce other model’s bias toward high-cardinality features by using weak learners and error-based reweighting, which results in more balanced SHAP feature importance scores^82^.In the summary plot (Fig. 9), each point represents a feature’s impact for a given sample, with color gradients indicating feature values, ranging from high to low.

Overall, topographical features are more influential than hydrological features (Fig. 9). Elevation has a major influence on model prediction. In general, surface runoff takes the path of the steepest descent, as it offers lesser resistance while flowing downhill. Infrastructural feature, distance to combined sewer outlet (STORMWATER), also stands out as a key contributor. In many U.S. cities, combined sewer systems handle both sewage and stormwater, a combination that can become hazardous even during moderate rainfall when the drainage capacity is exceeded, causing overflows. Roads closer to sewer outfalls experience faster drainage through shorter flow paths, reducing flood risk. Roads farther from outfalls face delayed drainage, potential backflow, and blockages, which increases likelihood of flooding during extreme storm events^50^. Curve number, determined by the soil type and ground cover of a location, indicates the potential for runoff generation. Curve number simplifies the complex interactions of hydrologic variables into a single number. Areas with higher curve number like road surfaces are highly susceptible to flood as they generate more surface runoff and less infiltration. Likewise, areas with lower curve number are less susceptible to flood as they have higher ability to absorb water. Urban areas, characterized by extensive impervious surfaces, produce greater runoff during rainfall events. In contrast, features like road slope and surface roughness have less impact on the model’s accuracy.

Rainfall plays a less prominent role in road flood predictions compared to topographic and infrastructural features. In urban environments, surface runoff is heavily influenced by the capacity and design of drainage systems. When rainfall exceeds the drainage infrastructure’s capacity, especially in areas served by combined sewer systems, it leads to surface water accumulation, regardless of the actual rainfall intensity. Even moderate rain can overwhelm these systems if they are undersized, poorly maintained, or obstructed, causing localized flooding. Urban elevation further compounds this issue. Low-lying areas and natural depressions tend to accumulate runoff, while higher elevations experience faster water movement due to gravity. In cities with significant elevation variations, stormwater from higher terrain flows onto lower-lying road networks, increasing the risk of flooding and disrupting transportation infrastructure. This persistence of flood-prone areas, regardless of rainfall intensity, highlights the dominant influence of topography and drainage infrastructure.

## Discussion

This study demonstrates that ensemble learning, particularly stacking, can enhance predictive performance of urban road network flooding compared to traditional base learners. Leveraging diverse model architectures through meta-learning reduces prediction uncertainty and improved robustness, especially when working with complex, nonlinear and data-limited urban systems. Our findings are consistent with previous studies^41,42^ showing the advantages of ensemble approaches in disaster risk prediction, where combining multiple learners helps address the limitations of a single model (e.g., overfitting, bias and instability). Unlike prior studies^6^ that primarily focused on riverine flood hazards, this work extends ensemble applications to pluvial flood prediction in highly urbanized areas. Crowd-sourced flood observations allowed models to capture localized disruptions often missed by FEMA floodplain maps. Comparison with FEMA’s designated 100-year and 500-year flood zones revealed that several high flood hazard road segments identified in our study fall outside mapped floodplain. This underscores the critical importance of incorporating surface flooding processes into urban hazard assessments.

The SHAP analysis further highlights that elevation is a dominant predictor, aligning with hydrologic principles of water accumulation in low-lying areas. Other conditioning factors, such as proximity to streams, sewer outfalls, and road surface characteristics contribute meaningfully to the classification. This suggests that integrating multiple geospatial and infrastructure attributes can improve fine-scale risk characterization, providing more actionable information for urban planners and emergency managers. Our results show that over 40% of energy and emergency services in Washington, D.C. are located within high-hazard road networks, which has critical implications for resilience planning. Road closures in these areas could hinder rapid response during flood events and amplify cascading disruptions across interdependent infrastructure systems.

Our study is subject to some limitations highlighting the need for future research. The crowd-sourced dataset, while valuable, is relatively small and may be biased toward heavily reported or densely populated areas. Expanding the dataset through systematic integration of traffic sensors, mobile applications, and high-resolution satellite imagery could improve model generalizability. This study primarily focuses on static geospatial predictors, whereas dynamic factors, such as temporal rainfall patterns and real-time drainage capacity, were not explicitly incorporated. Hybrid approaches that embed ensemble machine learning into hydrodynamic models may capture these processes more effectively. Future research should explore the scalability of ensemble frameworks to other metropolitan areas with diverse topographic and infrastructural contexts. Incorporating real-time data streams and advancing explainable AI techniques could further enhance model usability in decision-support systems. Our study primarily focuses on flood hazard prediction and the exposure of critical infrastructure to high-hazard zones. Future research could build on this work by integrating hazard, exposure, and vulnerability to provide a more comprehensive assessment of flood risk and its potential impacts.

## Conclusion

Our study demonstrates the effectiveness of ensemble machine learning for enhancing urban flood hazard prediction. By training both base and super ensemble learners on crowd-sourced flood datasets, we found that ensemble learners outperform traditional machine learning models, with Stacking achieving the highest accuracy (0.84), precision(0.82), and F1-score(0.82). AdaBoost and Gradient Boosting outperformed in recall(0.84) and ROC AUC(0.92). Hazard mapping revealed that 16.4% of DC road networks are in highly flood-prone zones, primarily concentrated in dense urban centers. FEMA 100-yr and 500-yr floodplain maps captured less than 20% of these segments. Additionally, a substantial portion of critical infrastructure is exposed to high flood hazard, with 66.7% of energy services and 44.4% of emergency services located along high-hazard road segments. Elevation emerges as the most influential predictor, followed by infrastructure-related factors such as proximity to combined sewer outlets.

These findings highlight the value of data-driven, fine-scale approaches for urban flood hazard assessment. By integrating ensemble learning with emerging data sources, city planners, transportation agencies, and policymakers can obtain more accurate hazard information to guide infrastructure investment, emergency response planning, and long-term resilience strategies. Such approaches can complement traditional mapping frameworks, offering actionable insights that help safeguard critical services, maintain mobility, and strengthen preparedness for extreme weather events.

## Data Availability

Code for model performance and feature importance is available at the GitHub repository (DC Road Flood).

## References

[1] Jacobs, J. M. et al. Chapter 12: Transportation. Impacts, Risks, and Adaptation in the United States: The Fourth National Climate Assessment, Vol. II. Tech. Rep., U.S. Global Change Research Program. 10.7930/NCA4.2018.CH12 (2018).

[2] He, Y. et al. Mobility and resilience: A global assessment of flood impacts on road transportation networks. *Policy Research Working Paper Series* (2022).

[3] Department of Transportation. How do weather events impact roads? (2024).

[4] Bates, P. D. et al. Combined modeling of US fluvial, pluvial, and coastal flood hazard under current and future climates. *Water Resour. Res.* **57**, e2020WR028673. 10.1029/2020WR028673 (2021).

[5] FEMA. Flood maps. https://msc.fema.gov (2024).

[6] Dey, H., Haque, M. M., Shao, W., VanDyke, M. & Hao, F. Simulating flood risk in Tampa Bay using a machine learning driven approach. *npj Nat. Hazards* **1**, 40 (2024).

[7] Dey, H., Shao, W., Haque, M. & VanDyke, M. Enhancing flood risk analysis in Harris County: Integrating flood susceptibility and social vulnerability mapping. *J. Geovisu. Spat. Anal.* **8**, 19 (2024).

[8] Shao, W. et al. Understanding the effects of past flood events and perceived and estimated flood risks on individuals’ voluntary flood insurance purchase behavior. *Water Res.* **108**, 391–400 (2017). 27876363 10.1016/j.watres.2016.11.021

[9] Nelson-Mercer, B., Kim, T., Tran, V. N. & Ivanov, V. Pluvial flood impacts and policyholder responses throughout the united states. *npj Nat. Hazards* **2**, 8 (2025).

[10] Alfieri, L. et al. Global projections of river flood risk in a warmer world. *Earths Future* **5**, 171–182 (2017).

[11] Kulp, S. A. & Strauss, B. H. New elevation data triple estimates of global vulnerability to sea-level rise and coastal flooding. *Nat. Commun.* **10**, 4844 (2019). 31664024 10.1038/s41467-019-12808-z PMC6820795

[12] Tonn, G. & Czajkowski, J. Evaluating the risk and complexity of pluvial flood damage in the us. *Water Econ. Policy* **8**, 2240002 (2022).

[13] Zahura, F. T. et al. Training machine learning surrogate models from a high-fidelity physics-based model: Application for real-time street-scale flood prediction in an urban coastal community. *Water Resour. Res.* **56**, e2019WR027038. 10.1029/2019WR027038 (2020).

[14] Hickmon, N. L., Varadharajan, C., Hoffman, F. M. & Wainwright, H. M. & S Collis. *Artificial Intelligence for Earth System Predictability* (AI4ESP) (2022).

[15] Wing, O. E. et al. Inequitable patterns of us flood risk in the Anthropocene. *Nat. Clim. Change* **12**, 156–162 (2022).

[16] Kendon, E. J. et al. Heavier summer downpours with climate change revealed by weather forecast resolution model. *Nat. Clim. Change* **4**, 570–576 (2014).

[17] Mirzaei, P. A. CFD modeling of micro and urban climates: Problems to be solved in the new decade. *Sustain. Cities Soc.* **69**, 102839. 10.1016/j.scs.2021.102839 (2021).

[18] Gangrade, S. et al. Unraveling the 2021 central Tennessee flood event using a hierarchical multi-model inundation modeling framework. *J. Hydrol.* **625**, 130157 (2023).

[19] Koenig, T. A. et al. *Identifying and preserving high-water mark data*. Tech. Rep., US Geological Survey (2016).

[20] Ginkel, K. C., Dottori, F., Alfieri, L., Feyen, L. & Koks, E. E. Flood risk assessment of the European road network. *Nat. Hazard.* **21**, 1011–1027 (2021).

[21] Sentinel. ESA Standard Document.

[22] Safaei-Moghadam, A., Tarboton, D. & Minsker, B. Estimating the likelihood of roadway pluvial flood based on crowdsourced traffic data and depression-based dem analysis. *Nat. Hazards Earth Syst. Sci.* **23**, 1–19 (2023).

[23] Walker, C. L. et al. Towards development of a roadway flood severity index. *Transp. Res. Interdiscip. Perspect.* **27**, 101218 (2024).

[24] Puttinaovarat, S. & Horkaew, P. Flood forecasting system based on integrated big and crowdsource data by using machine learning techniques. *IEEE Access* **8**, 5885–5905. 10.1109/ACCESS.2019.2963819 (2020) (**Conference Name: IEEE Access.**).

[25] Nevo, S. et al. Flood forecasting with machine learning models in an operational framework. *Hydrol. Earth Syst. Sci.* **26**, 4013–4032. 10.5194/hess-26-4013-2022 (2022).

[26] Mehedi, M. A. A., Smith, V., Hosseiny, H. & Jiao, X. Unraveling the complexities of urban fluvial flood hydraulics through AI. *Sci. Rep.* **12**, 18738. 10.1038/s41598-022-23214-9 (2022). 36333429 10.1038/s41598-022-23214-9 PMC9636396

[27] Bhattarai, Y., Duwal, S., Sharma, S. & Talchabhadel, R. Leveraging machine learning and open-source spatial datasets to enhance flood susceptibility mapping in transboundary river basin. *Int. J. Digit. Earth* **17**, 2313857. 10.1080/17538947.2024.2313857 (2024).

[28] Jin, H. et al. An intelligent framework for spatiotemporal simulation of flooding considering urban underlying surface characteristics. *Int. J. Appl. Earth Obs. Geoinf.* **130**, 103908. 10.1016/j.jag.2024.103908 (2024).

[29] Bhattarai, Y., Bista, S., Talchabhadel, R., Duwal, S. & Sharma, S. Rapid prediction of urban flooding at street-scale using physics-informed machine learning-based surrogate modeling. *Total Environ. Adv.* **12**, 200116. 10.1016/j.teadva.2024.200116 (2024).

[30] Liang, Z. et al. Graph spiking neural network for advanced urban flood risk assessment. *iScience* **27**, 111037. 10.1016/j.isci.2024.111037 (2024). 39524329 10.1016/j.isci.2024.111037 PMC11544073

[31] Dong, S., Yu, T., Farahmand, H. & Mostafavi, A. Probabilistic modeling of cascading failure risk in interdependent channel and road networks in urban flooding. *Sustain. Cities Soc.* **62**, 102398. 10.1016/j.scs.2020.102398 (2020).

[32] Abu-Salih, B. et al. The development of a road network flood risk detection model using optimised ensemble learning. *Eng. Appl. Artif. Intell.* **122**, 106081. 10.1016/j.engappai.2023.106081 (2023).

[33] Ha, H. et al. Flash flood susceptibility prediction mapping for a road network using hybrid machine learning models. *Nat. Hazards* **109**, 1247–1270. 10.1007/s11069-021-04877-5 (2021).

[34] Brown, G., Wyatt, J., Harris, R. & Yao, X. Diversity creation methods: A survey and categorisation. *Inf. Fusion* **6**, 5–20. 10.1016/j.inffus.2004.04.004 (2005).

[35] Zounemat-Kermani, M., Batelaan, O., Fadaee, M. & Hinkelmann, R. Ensemble machine learning paradigms in hydrology: A review. *J. Hydrol.* **598**, 126266. 10.1016/j.jhydrol.2021.126266 (2021).

[36] Paisitkriangkrai, S., Shen, C. & Hengel, A. Pedestrian detection with spatially pooled features and structured ensemble learning. *IEEE Trans. Pattern Anal. Mach. Intell.* **38**, 1243–1257. 10.1109/TPAMI.2015.2474388 (2016). 26336118 10.1109/TPAMI.2015.2474388

[37] Mienye, I. D., Sun, Y. & Wang, Z. Improved predictive sparse decomposition method with densenet for prediction of lung cancer. *Int. J. Comput.* **1**, 533–541. 10.47839/ijc.19.4.1986 (2020).

[38] Tyralis, H., Papacharalampous, G. & Langousis, A. Super ensemble learning for daily streamflow forecasting: Large-scale demonstration and comparison with multiple machine learning algorithms. *Neural Comput. Appl.* **33**, 3053–3068. 10.1007/s00521-020-05172-3 (2021).

[39] Simafranca, N. et al. Modeling wildland fire burn severity in California using a spatial Super Learner approach. *Environ. Ecol. Stat.* **31**, 387–408. 10.1007/s10651-024-00601-1 (2024).

[40] Valdes, G., Interian, Y., Gennatas, E. & Laan, M. The conditional super learner. *IEEE Trans. Pattern Anal. Mach. Intell.* **44**, 10236–10243. 10.1109/TPAMI.2021.3131976 (2022). 34851823 10.1109/TPAMI.2021.3131976

[41] Yuan, F. et al. Predicting road flooding risk with crowdsourced reports and fine-grained traffic data. *Comput. Urban Sci.* **3**, 15. 10.1007/s43762-023-00082-1 (2023).

[42] Safaei-Moghadam, A., Hosseinzadeh, A. & Minsker, B. Predicting real-time roadway pluvial flood risk: A hybrid machine learning approach coupling a graph-based flood spreading model, historical vulnerabilities, and Waze data. *J. Hydrol.* **637**, 131406. 10.1016/j.jhydrol.2024.131406 (2024).

[43] Castro, U. et al. Towards smart mobility during flooding events in urban areas using crowdsourced information. In *2019 IEEE International Smart Cities Conference (ISC2)* 154–159. 10.1109/ISC246665.2019.9071781 (2019).

[44] Zhang, Y., Ayyub, B. M., Zhang, D., Huang, H. & Saadat, Y. Impact of water level rise on urban infrastructures: Washington, DC, and Shanghai as case studies. *Risk Anal.* **39**, 2718–2731. 10.1111/risa.13390 (2019). 31441948 10.1111/risa.13390

[45] NOAA. Flooding in Washington, D.C. (2025).

[46] U.S. Army Corps of Engineers. Metropolitan Washington District of Columbia Coastal Storm Risk Management Feasibility Study (2022).

[47] Environmental Systems Research Institute. World_basemap_v2 vector tile service. https://basemaps.arcgis.com/arcgis/rest/services/World_Basemap_v2/VectorTileServer (2025).

[48] Environmental Systems Research Institute. *ArcGIS Pro 3.4.0*. ESRI (2024).

[49] Street, F. Federal Triangle, DC Flood Map and Climate Risk Report (2025).

[50] Greeley and Hansen LLC. Federal Triangle Stormwater Drainage Study. *National Capital Planning Comission* (2011).

[51] Fan, C., Jiang, X. & Mostafavi, A. A network percolation-based contagion model of flood propagation and recession in urban road networks. *Sci. Rep.* **10**, 13481 (2020). 32778733 10.1038/s41598-020-70524-x PMC7417581

[52] Yuan, F., Xu, Y., Li, Q. & Mostafavi, A. Spatio-temporal graph convolutional networks for road network inundation status prediction during urban flooding. *Comput. Environ. Urban Syst.* **97**, 101870. 10.1016/j.compenvurbsys.2022.101870 (2022).

[53] Löwe, R., Böhm, J., Jensen, D. G., Leandro, J. & Rasmussen, S. H. U-FLOOD—Topographic deep learning for predicting urban pluvial flood water depth. *J. Hydrol.* **603**, 126898. 10.1016/j.jhydrol.2021.126898 (2021).

[54] Piadeh, F. et al. Event-based decision support algorithm for real-time flood forecasting in urban drainage systems using machine learning modelling. *Environ. Model. Softw.* **167**, 105772. 10.1016/j.envsoft.2023.105772 (2023).

[55] Zhang, J. et al. Multi-radar multi-sensor (MRMS) quantitative precipitation estimation: Initial operating capabilities. *Bull. Am. Meteor. Soc.*10.1175/BAMS-D-14-00174.1 (2016).

[56] MRMS. Multi-Radar/Multi-Sensor System (MRMS).

[57] Dewitz, J. National Land Cover Database (NLCD) 2021 Products. 10.5066/P9JZ7AO3 (2023).

[58] Islam, A. R. M. T. et al. Flood susceptibility modelling using advanced ensemble machine learning models. *Geosci. Front.* **12**, 101075. 10.1016/j.gsf.2020.09.006 (2021).

[59] Soil Conservation Service. National engineering handbook, section 4: Hydrology (1985).

[60] NOAA. Soil data (2024).

[61] Gañan-Cardenas, E., Pemberthy-R, J. I., Suárez-Gómez, M. L., Ballesteros, J. R. & Branch-Bedoya, J. W. Machine learning framework for predicting urban road speed profiles and uncertainty. *Int. J. Transp. Sci. Technol.* (2025).

[62] District of Columbia, Chief Data Officer. Open data DC (2025).

[63] Earth Resources Observation and Science (EROS) Center. National land cover database (nlcd). Accessed 11 Aug 2025 (2018)

[64] NOAA. Multi-radar multi-sensor (mrms) system. Accessed 11 Aug 2025 (2025).

[65] Tehrany, M. S., Pradhan, B. & Jebur, M. N. Flood susceptibility mapping using a novel ensemble weights-of-evidence and support vector machine models in GIS. *J. Hydrol.* **512**, 332–343. 10.1016/j.jhydrol.2014.03.008 (2014).

[66] Mangkhaseum, S., Bhattarai, Y., Duwal, S. & Hanazawa, A. Flood susceptibility mapping leveraging open-source remote-sensing data and machine learning approaches in Nam Ngum River Basin (NNRB), Lao PDR. *Geomat. Nat. Hazards Risk* **15**, 2357650 (2024) (**Publisher: Taylor and Francis.**).

[67] Breiman, L. Random forests. *Mach. Learn.* **45**, 5–32. 10.1023/A:1010933404324 (2001).

[68] Hosseiny, H., Nazari, F., Smith, V. & Nataraj, C. A framework for modeling flood depth using a hybrid of hydraulics and machine learning. *Sci. Rep.* **10**, 8222. 10.1038/s41598-020-65232-5 (2020). 32427970 10.1038/s41598-020-65232-5 PMC7237697

[69] Schapire, R. E. Explaining AdaBoost. In *Empirical Inference* (eds. Schölkopf, B., Luo, Z. & Vovk, V.) 37–52 (Springer, 2013). 10.1007/978-3-642-41136-6_5.

[70] scikit-learn. Scikit-learn Developers (2018). Publication Title: scikit-learn.

[71] Osamor, V. C. & Okezie, A. F. Enhancing the weighted voting ensemble algorithm for tuberculosis predictive diagnosis. *Sci. Rep.* **11**, 14806 (2021). 34285324 10.1038/s41598-021-94347-6 PMC8292494

[72] Wolpert, D. H. Stacked generalization. *Neural Netw.* **5**, 241–259 (1992).

[73] Wolpert, D. H., Macready, W. G. et al. Combining stacking with bagging to improve a learning algorithm. *Santa Fe Inst. Tech. Rep.* **30** (1996).

[74] Wang, Q. & Lu, H. A novel stacking ensemble learner for predicting residual strength of corroded pipelines. *npj Mater. Degrad.* **8**, 87 (2024).

[75] Loon, W., Fokkema, M., Szabo, B. & Rooij, M. Stacked penalized logistic regression for selecting views in multi-view learning. *Inf. Fusion* **61**, 113–123 (2020).

[76] Friedman, J. H., Hastie, T. & Tibshirani, R. Regularization paths for generalized linear models via coordinate descent. *J. Stat. Softw.* **33**, 1–22 (2010). 20808728 PMC2929880

[77] Bui, D. T. et al. Novel hybrid evolutionary algorithms for spatial prediction of floods. *Sci. Rep.* **8**, 15364 (2018) (**Publisher: Nature Publishing Group UK London.**). 30337603 10.1038/s41598-018-33755-7 PMC6193992

[78] Razavi-Termeh, S. V., Sadeghi-Niaraki, A., Abba, S. I., Hussain, J. & Choi, S.-M. Flood-prone area mapping using a synergistic approach with swarm intelligence and gradient boosting algorithms. *Sci. Rep.* **15**, 27924 (2025). 40745431 10.1038/s41598-025-12022-6 PMC12314035

[79] DCgov. Open Data DC.

[80] Abdollahi, A. & Pradhan, B. Explainable artificial intelligence (XAI) for interpreting the contributing factors feed into the wildfire susceptibility prediction model. *Sci. Total Environ.* **879**, 163004. 10.1016/j.scitotenv.2023.163004 (2023). 36965733 10.1016/j.scitotenv.2023.163004

[81] Pradhan, B., Lee, S., Dikshit, A. & Kim, H. Spatial flood susceptibility mapping using an explainable artificial intelligence (XAI) model. *Geosci. Front.* **14**, 101625. 10.1016/j.gsf.2023.101625 (2023).

[82] Wyner, A. J., Olson, M., Bleich, J. & Mease, D. Explaining the success of adaboost and random forests as interpolating classifiers. *J. Mach. Learn. Res.* **18**, 1–33 (2017).

